# Postoperative Fever: The Potential Relationship with Prognosis in Node Negative Breast Cancer Patients

**DOI:** 10.1371/journal.pone.0015903

**Published:** 2010-12-29

**Authors:** Tingting Yan, Wenjin Yin, Liheng Zhou, Yiwei Jiang, Zhenzhou Shen, Zhimin Shao, Jinsong Lu

**Affiliations:** 1 Department of Breast Surgery, Fudan University Shanghai Cancer Center, Shanghai, China; 2 Department of Oncology, Shanghai Medical College, Fudan University, Shanghai, China; The University of Hong Kong, China

## Abstract

**Background:**

Postoperative fever may serve as an indirect sign to reflect the alterations of the host milieu caused by surgery. It still remains open to investigation whether postoperative fever has a bearing on prognosis in patients with lymph node negative breast cancers.

**Methods:**

We performed a retrospective study of 883 female unilateral patients with lymph node negative breast cancer. Fever was defined as an oral temperature ≥38 in one week postoperation. Survival curves were performed with Kaplan-Meier method, and annual relapse hazard was estimated by hazard function.

**Findings:**

The fever patients were older than those without fever (*P*<0.0001). Hypertensive patients had a propensity for fever after surgery (*P* = 0.011). A statistically significant difference was yielded in the incidence of fever among HR+/ERBB2-, ERBB2+, HR-/ERBB2- subgroups (*P* = 0.012). In the univariate survival analysis, we observed postoperative fever patients were more likely to recur than those without fever (*P* = 0.0027). The Cox proportional hazards regression analysis showed that postoperative fever (*P* = 0.044, RR = 1.89, 95%CI 1.02–3.52) as well as the HR/ERBB2 subgroups (*P* = 0.013, HR = 1.60, 95%CI 1.09–2.31) was an independent prognostic factor for relapse-free survival.

**Conclusion:**

Postoperative fever may contribute to relapse in node negative breast cancer patients, which suggests that changes in host milieu related to fever might accelerate the growth of micro-metastatic foci. It may be more precise to integrate both tumor- and host-related factors for the evaluation of relapse risk.

## Introduction

Worldwide, more than a million women are diagnosed with breast cancer every year, accounting for 27% of all female new cases in the United States in 2009 [1]. The incidence rates of breast cancer vary considerably, with the highest rates in the North America and the lowest rates in Africa and Asia. However, the number of new breast cancer cases has risen dramatically in developing countries, especially in China over recent years [1], [2], which mainly results from the aging of society and changes of lifestyle. On the other hand, the development of imaging techniques also contributed to the increased diagnosis of earlier breast cancer patients, most of whom were featured by negative axillary lymph node (ALN) [3], [4], [5].

Women with ALN negative breast cancers have a relatively good prognosis. However, approximately 20% of these patients will experience disease recurrence and subsequent death, even if some of them may benefit from adjuvant systemic therapy [6]. Nowadays, various guidelines are available for risk allocation to determine the optimal therapeutic modality in node-negative women [7], [8]. Over the past years, a number of clinicopathological parameters have been regarded as potential prognosticators in patients with early-stage node-negative breast cancer, exemplified by tumor size, histological grade, cathepsin-D status, p53 mutation, ERBB2 overexpression, Ki-67 index, S-phase fraction, mitotic index, and vascular invasion *etc*. [6], [9], [10]. Besides, some multigene assays have also been validated to provide useful information for women with node-negative breast cancers [10], [11], [12], [13]. The 21-gene Recurrence Score (RS) assay (Oncotype DX, Genomic Health, Redwood city, CA) is one of the best tools along with the pathology to help quantify the risk of distant recurrence as well as predict the magnitude of chemotherapy benefit in tamoxifen-treated patients with lymph node-negative, estrogen receptor(ER)-positive breast cancer [11], [13], [14]. Unfortunately, all the factors above are confined to tumor biology *per se* and have been described in an extensive body of literature, while the impact of the host's internal environments on clinical outcomes still remains unclear and open to investigation.

It has long been recognized that the development and progression of a certain cancer is the consequence of dynamic and reciprocal interaction between tumor cells and their micro-environments. Two centuries ago Paget proposed the “seed and soil” hypothesis, which states that specific organs (the “soil”) harbor metastases from selected cancer cells (the “seeds”) [15]. The potential of tumor cells to colonize and propagate depends on their cross-talk with the extracellular matrix components of the distant organ. By this token, what can influence the host milieu may somewhat modify the course of the disease. Surgery, as a stimulus to the host, has been demonstrated at least partially to be responsible for such effect. Manifold studies revealed that primary tumor removal might lead to sudden acceleration of metastatic process by releasing the growth factor into the blood [16], which indicated that surgical extirpation is not just a local phenomenon without any other biological consequences. Accordingly, a better knowledge of host responses induced by surgery may contribute to a fuller perspective of prognostic profiles in breast cancer patients.

Postoperative increased core temperature is one of the host responses to surgery. Despite its prevalence in postoperative patients, the logics behind it are still poorly understood. The most frequently observed etiology of fever after surgery is related to the normal thermoregulatory response without infection [17]. Therefore, postoperative fever may serve as an indirect sign to reflect the alterations of the host milieu caused by surgery.

On these premises, we sought to probe into the possible correlation between postoperative fever and survival times. Nowadays, few studies have been conducted to bring it to light in breast cancer patients. However, only one small study observed that the relative risk of recurrence after postoperative fever increased significantly [18]. That study retrospectively included 378 breast cancer patients of all stages, which failed to mirror the true picture of postoperative fever since its role might be shadowed by sufficiently established prognostic factors such as ALN status. Accordingly, a retrospective analysis was carried out among ALNs-negative breast cancer patients undergoing surgery in Fudan University Shanghai Cancer Center, Shanghai, China, offering the implications for the underlying mechanism.

## Methods

### Patients

A total of 883 node-negative breast cancer patients were selected retrospectively from a large database of patients who underwent surgery between January 1, 2000 and December 31, 2002 in Fudan University Shanghai Cancer Center, Shanghai, China. Before surgery, such evaluations were mandatory for each patient as complete physical examination, chest radioscopy, bilateral mammography, ECG, ultrasonography of breasts, aillary fossa, cervical parts, abdomen and pelvis, complete blood count, and routine biochemical tests to make an exact staging. Each patient received lumpectomy or mastectomy followed by adjuvant therapy according to the guidelines or recommendations used at the time of surgery. Patients with bilateral primary breast cancer and/or with initial systemic metastases were excluded from the study. The body temperature was measured with an oral thermometer three times a day for each patient. A fever was defined as one measured oral temperature ≥38°C in one week postoperation, which was similar to other relevant reports [19], [20].

Follow-up information regarding tumor recurrence and survival status was accomplished through the retrieve of medical records kept in the outpatient department, personal contact with the patient as well as the assistance of Shanghai Center for Disease Control and Prevention (CDC). Personal contact with the patient referred to routine correspondence or telephone visits, which were carried out in Fudan University Shanghai Cancer Center every 3 months during the first two years, every 6 months during the next two years and once a year thereafter. Recurrence or its absence was diagnosed by query to the patient, by biopsy, or by scan of bone, chest, abdomen, pelvis or skull. Whenever the tumor recurred, additional information, including sites of recurrence and therapy, was requested.

Ethical appraisal from Medical Ethics Committee at Fudan University Shanghai Cancer Center was exempt for the retrospective study involving the collection or analysis of existing data in accordance with federal regulations 45 CFR 46 [21]. And therefore, written consent given by the patients was not needed for their hospitalization and follow-up information to be stored in the center database and used for research. Furthermore, we only took anonymous data from this database and the Ethics Committee waived the need for approval and consent.

### Immunohistochemistry

Immunohistochemical staining was carried out as a standard operating procedure in the pathology department of Fudan University Shanghai Cancer Center. All primary monoclonal antibodies for estrogen receptor (ER), progesterone receptor (PR) and ERBB2 were from Dako. The staining results were assessed by at least two pathologists, using a semiquantitative scoring system, where integrated the proportion score and the intensity score. The proportion score, indicating the percentage of tumor cells stained, was interpreted such that a score of 0 required no staining seen, 1 required ≤25% of cells positive, 2 required 25–50% of cells stained, 3 required 50–75% of positive cells and 4 required >75% of staining cells. As to the intensity score, a negative result was defined as a score of 0, weakly positive as 1, moderately positive as 2, and strongly positive as 3. The final score was calculated as the product of the proportion score and the intensity score. Thereby, staining results ranged from score 0 to 12.The scoring system for ER and PR was defined as negative for score 0 and positive for scores of 1∼12 with the nucleic staining of carcinoma cells, whereas ERBB2 was defined as negative for scores of 0∼8 (namely, 0, 1+ and 2+ in the DAKO scoring system) and positive for strong membranous staining with scores of 9∼12 (namely DAKO score 3+).

### Statistical analysis

Relapse-free survival (RFS) was defined as the time from surgery to the earliest occurrence of recurrence (loco-regional or distant) or death from any cause. Those without any evidence of relapse were censored at the last date they were known to be alive. Clinicopathological parameters were compared between different subgroups using student's *t* test for continuous variables and chi-square test for categorical variables.

Survival curves were performed by the Kaplan–Meier method and were compared using the log-rank test. Multivariate Cox proportional hazards regression analysis was applied to modeling the relationship between postoperative fever and RFS, adjusted for age (≤50 years, >50 years), tumor size (≤5 cm, >5 cm), history of hypertension (yes, no), type of surgery (mastectomy, breast conservative surgery) as well as the hormone receptor HR/ERBB2 subgroups (HR+/ERBB2-, ERBB2+, HR-/ERBB2- or triple negative). Relative risks (RRs) were presented with their 95% confidence intervals (CIs). For graphical display of RFS, annual hazard rates were estimated using a Kernel method of smoothing. All the analyses were considered statistically significant when the *P*-value was less than 0.05. All statistical analyses were performed with Stata statistical software package (release 10.0; Stata Corporation, College Station, Texas, USA).

## Results

### General characteristics

The median age of the 883 patients was 52 years old (range 25∼91) at diagnosis. Among the study population, 230 patients (26.05%) suffered from postoperative fever. The patients with postoperative fever tended to be older than those without fever (55.07±11.56 vs. 51.38±11.23, *P*<0.0001). ERBB2+ patients were more likely to develop postoperative fever than HR+/ERBB2- and triple negative subgroup (42.62%, 21.71% and 27.41% respectively, *P* = 0.003). There was a significantly increased incidence of postoperative fever in hypertensive patients than those with normal blood pressure (33.00% v*s*. 24.01%, *P* = 0.011). In addition, such was likewise the case for the patients undergoing mastectomy and breast conservation surgery (27.47% *vs.* 12.20%, *P* = 0.003). When compared to patients without fever after surgery, more relapse events were observed in those who have postoperative fever (*P* = 0.004) with a median follow-up of 43 months (range 3–90 months) (Table 1).

**Table 1:** Summary of patient's characteristics and its association with postoperative fever.

Variable	Univariate analysis, n(%)		*P* value
	Fever	No fever	
Mean Age at Diagnosis (year, x±SD)	55.07±11.56	51.38±11.23	<0.0001
Age at diagnosis≤50 years>50 years	79(34.35)151(65.65)	365(55.90)288(44.10)	<0.0001
Tumor size≤5 cm>5 cm	222 (96.52)8(3.48)	630 (96.48)23(3.52)	0.975
HR/ERBB2 SubgroupHR+/ERBB2−ERBB2+HR-/ERBB2−Unknown	66(28.70)26(11.30)111(48.26)27(11.74)	238(36.45)35(5.36)294(45.02)86(13.17)	0.003
Type of SurgeryBreast conservation surgerymastectomy	10(4.35)220(95.65)	72(11.03)581(88.97)	0.003
HypertensionYesNo	66(28.70)164(71.30)	134(20.52)519(79.48)	0.011
Relapse eventsYesNo	21(9.13)209(90.87)	27(4.13)626(95.87)	0.004

### Survival analysis

**Figure 1:**
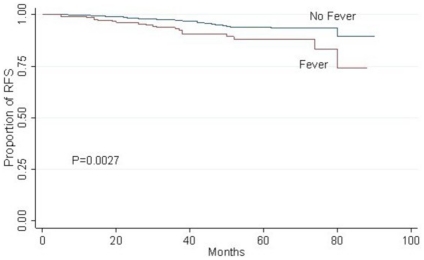
Kaplan–Meier curves for RFS in 883 ALN negative breast cancer patients by postoperative fever.

**Figure 2:**
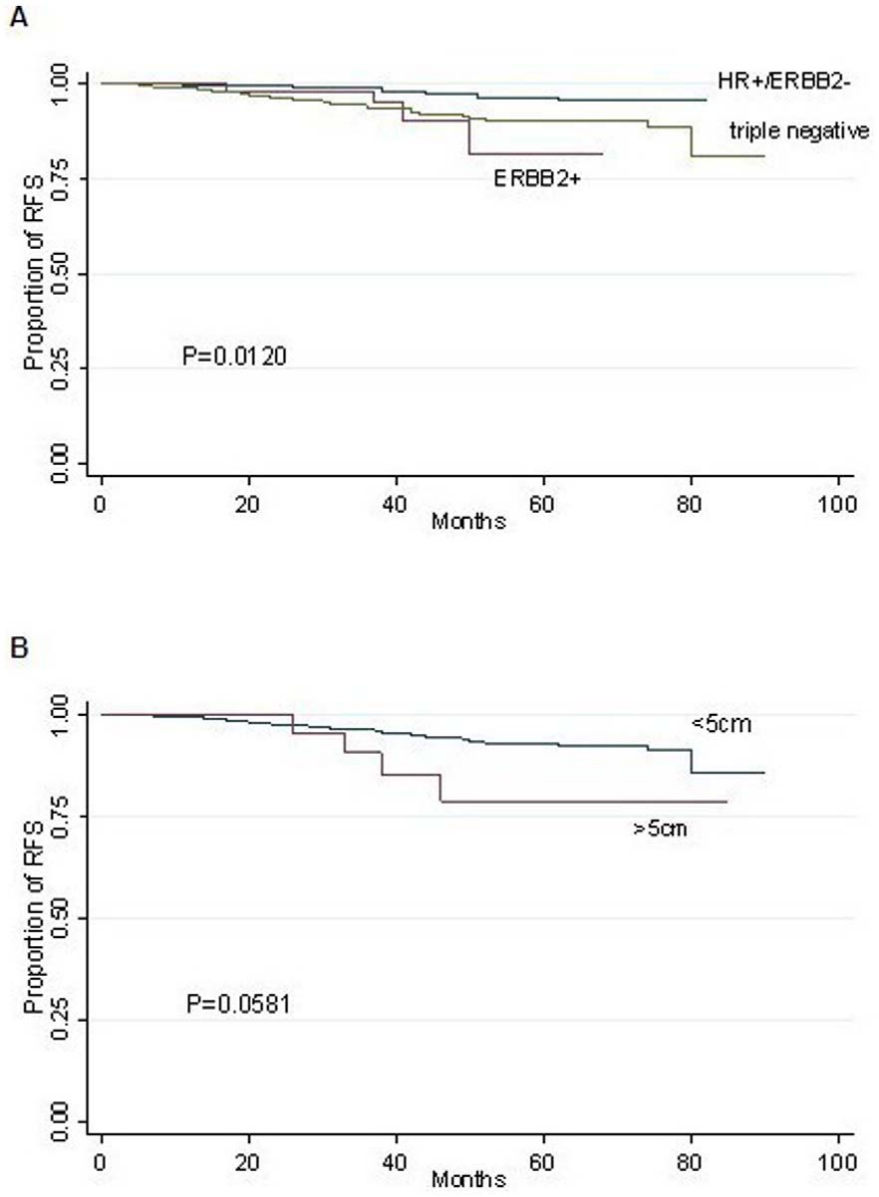
Kaplan–Meier curves for RFS in 883 ALN negative breast cancer patients by HR/ERBB2(A) and tumor size (B).

In the univariate survival analysis, we found significantly different RFS between patients with and without postoperative fever (*P* = 0.0027) (Figure 1), with the rate of 88.18% for the former and 93.94% for the later at the 5th year. Besides, a similar effect was also noted among different HR/ERBB2 subgroups (*P* = 0.012) (Figure 2A), while marginal significance was achieved between patients with larger and smaller tumors (*P* = 0.0581) (Figure 2B).

In Cox proportional hazards regression analysis, we found that HR/ERBB2 subgroup (*P* = 0.013; RR = 1.60; 95%CI 1.90–2.31) and fever (*P* = 0.044; RR = 1.89; 95%CI 1.02–3.52) were independent prognostic factors for RFS (Table 2).

**Table 2:** Multivariate Cox proportional hazards regression analysis for RFS in 883 ALN negative breast cancer patients.

Variable	RR	95% CI of RR	*P* value
Fever	1.89	1.02–3.52	0.044
Age(≤50 *vs*.>50)	1.77	0.89–3.50	0.102
Type of surgery	0.49	0.19–1.29	0.148
Hypertension	0.66	0.31–1.38	0.268
Tumor size(≤5 cm *vs*.>5 cm)	2.19	0.67–7.18	0.194
HR/ERBB2 subgroup	1.60	1.09–2.31	0.013

### Relapse hazard analysis

**Figure 3:**
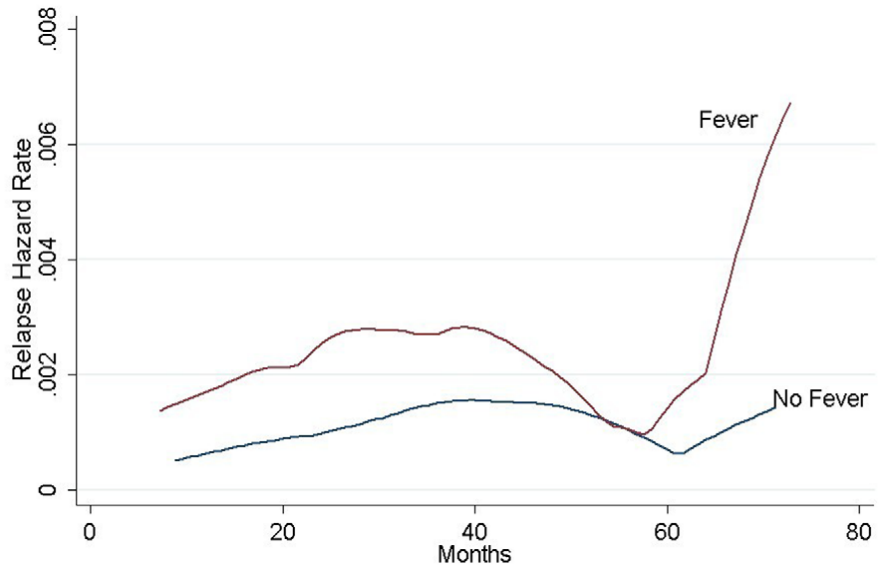
Annual relapse hazard rate for 883 ALN negative breast cancer patients by postoperative fever.

As to hazard peaks, discrepancies existed in different subgroups. The hazard plots displayed the long-term benefit in risk reduction for women without fever over those suffering from fever throughout the entire follow-up period. Patients with postoperative fever exhibited a modest increase from 25 to 40 months, whereas the hazard rate for those without fever showed an early major relapse surge peaking at the 45th month after surgery (Figure 3).

## Discussion

Our study is, to the best of our knowledge, the first and the largest retrospective analysis on the relationship between postoperative fever and prognosis in node negative breast cancer patients. Following breast cancer surgery, various complications arise, such as flap necrosis, infection, seroma formation, fever. Murthy and colleagues [22] reported that there was an increased risk of systemic recurrence in patients with wound problems at primary surgery, which indicated that host responses to the surgery, whether localized or systemic, might play an important role in clinical outcomes. However, such studies were almost confined to surgical incision complications while excluding postoperative fever.

As a complex systemic defense reaction of the host, fever refers to a rise in core temperature with resultant release of specific cytokines induced by infection, injury, surgical and general anesthesia trauma [23], [24], [25], [26]. Cytokines have been implicated as pivotal mediators of the pyrogenic or antipyretic process, including the pro-inflammatory tumor necrosis factor α (TNF-α), interleukin-6 (IL-6) and interleukin-1 (IL-1) as well as the anti-inflammatory interleukin-1 receptor antagonist (IL-1ra) and interleukin-10 (IL-10) [27]. The levels of cytokines with opposite function decide the occurrence and magnitude of fever and *vice versa*. Surgical and anaesthetic stress may elicit the imbalance between the pro- and anti- inflammatory cytokines leading to fever [27]. A large body of reports demonstrated the stimulatory role for TNF-α, IL-1 and IL-6 in the proliferation of breast tumors [28], [29], [30]. Therefore, the elevated expressions of these pro-inflammatory cytokines might cooperate to exacerbate the pre-existing subclinical lesions and accordingly promote recurrence and death in patients with postoperative fever, which might elucidate the detrimental effect of fever on prognosis in this analysis as well as other relevant reports [18], [31].

On the other hand, cytokines may also confer the resistance to routine systemic treatment. In *in vitro* studies, the autocrine production of IL-6 by tumor cells increased the resistance to chemotherapy [32]. What's more important, Zhang and coworkers [33] detected significantly higher serum IL-6 levels in patients unresponsive to chemo-endocrine therapy than the responsive counterparts (*P* = 0.0007) in metastatic breast cancer patients. Besides, higher IL-6 level was also associated with poorer survival. These data indicate that such cytokines may affect the responsiveness of cancer cells to pharmaceutical agents, which conduce to the generation of drug-resistant strains, constituting the origin of recurrence. On the basis of the findings above, these cytokines bridge the fever after surgery and the progression of cancer in a direct and indirect way, which may serve as novel targets in cancer therapy.

The study has some potential limitations because it is a retrospective study. For a substantial portion of the patients in this database, relpases are probably somewhat underreported or misinformed; however, underreporting or misinformation of relapses would have not varied by clinicopathological parameters. Because of the retrospective nature of this study, our findings are for hypothesis generation only and need to be confirmed in large prospective studies.

Our results suggest that postoperative fever is associated with an increased rate of relapse in primary breast cancer patients. Apart from conventional prognosticators including ALN status, postoperative fever may also be a pivotal indicator in terms of host response, which will help to improve the accuracy of predicting prognosis and discover a new strategy of treatment.

## References

[1] Jemal A, Siegel R, Ward E, Hao Y, Xu J (2009). Cancer statistics, 2009.. CA Cancer J Clin.

[2] (2002). Cancer incidence in five continents..

[3] Fracheboud J, Otto SJ, van Dijck JA, Broeders MJ, Verbeek AL (2004). Decreased rates of advanced breast cancer due to mammography screening in The Netherlands.. Br J Cancer.

[4] Schootman M, Jeffe D, Reschke A, Aft R (2004). The full potential of breast cancer screening use to reduce mortality has not yet been realized in the United States.. Breast Cancer Res Treat.

[5] Vacek PM, Geller BM, Weaver DL, Foster RS (2002). Increased mammography use and its impact on earlier breast cancer detection in Vermont, 1975-1999.. Cancer.

[6] Bull SB, Ozcelik H, Pinnaduwage D, Blackstein ME, Sutherland DA (2004). The combination of p53 mutation and neu/erbB-2 amplification is associated with poor survival in node-negative breast cancer.. J Clin Oncol.

[7] National Comprehensive Cancer Network:NCCN Clinical Practice Guidelines in Oncology: Breast Cancer (Version 2. 2007).. http://wwwnccnorg.

[8] Goldhirsch A, Ingle JN, Gelber RD, Coates AS, Thurlimann B (2009). Thresholds for therapies: highlights of the St Gallen International Expert Consensus on the primary therapy of early breast cancer 2009.. Ann Oncol.

[9] Mirza AN, Mirza NQ, Vlastos G, Singletary SE (2002). Prognostic factors in node-negative breast cancer: a review of studies with sample size more than 200 and follow-up more than 5 years.. Ann Surg.

[10] van de Vijver MJ, He YD, van't Veer LJ, Dai H, Hart AA (2002). A gene-expression signature as a predictor of survival in breast cancer.. N Engl J Med.

[11] Paik S, Shak S, Tang G, Kim C, Baker J (2004). A multigene assay to predict recurrence of tamoxifen-treated, node-negative breast cancer.. N Engl J Med.

[12] van't Veer LJ, Paik S, Hayes DF (2005). Gene expression profiling of breast cancer: a new tumor marker.. J Clin Oncol.

[13] Habel LA, Shak S, Jacobs MK, Capra A, Alexander C (2006). A population-based study of tumor gene expression and risk of breast cancer death among lymph node-negative patients.. Breast Cancer Res.

[14] Paik S, Tang G, Shak S, Kim C, Baker J (2006). Gene expression and benefit of chemotherapy in women with node-negative, estrogen receptor-positive breast cancer.. J Clin Oncol.

[15] Fidler IJ (2003). The pathogenesis of cancer metastasis: the ‘seed and soil’ hypothesis revisited.. Nat Rev Cancer.

[16] Fisher B, Gunduz N, Coyle J, Rudock C, Saffer E (1989). Presence of a growth-stimulating factor in serum following primary tumor removal in mice.. Cancer Res.

[17] Pile JC (2006). Evaluating postoperative fever: a focused approach.. Cleve Clin J Med.

[18] Teucher G, Schindler AE (1987). [Postoperative fever and prognosis in breast cancer].. Arch Geschwulstforsch.

[19] Michael Marcy S, Kohl KS, Dagan R, Nalin D, Blum M (2004). Fever as an adverse event following immunization: case definition and guidelines of data collection, analysis, and presentation.. Vaccine.

[20] Mitchell JD, Grocott HP, Phillips-Bute B, Mathew JP, Newman MF (2007). Cytokine secretion after cardiac surgery and its relationship to postoperative fever.. Cytokine.

[21] Explanation of Federal Regulations for Human Subjects Research, Part 1” Online Ethics Center for Engineering 9/11/2006 National Academy of Engineering Accessed: Tuesday, July 13, 2010..

[22] Murthy BL, Thomson CS, Dodwell D, Shenoy H, Mikeljevic JS (2007). Postoperative wound complications and systemic recurrence in breast cancer.. Br J Cancer.

[23] Kumar S, Mehta Y, Vats M, Chand R, Kapoor P (2007). An observational study to know the association of leukocytosis and fever with infection in post cardiac surgery patients.. Indian Heart J.

[24] Turek Z, Sykora R, Matejovic M, Cerny V (2009). Anesthesia and the microcirculation.. Semin Cardiothorac Vasc Anesth.

[25] McCarter MD, Mack VE, Daly JM, Naama HA, Calvano SE (1998). Trauma-induced alterations in macrophage function.. Surgery.

[26] Majetschak M, Borgermann J, Waydhas C, Obertacke U, Nast-Kolb D (2000). Whole blood tumor necrosis factor-alpha production and its relation to systemic concentrations of interleukin 4, interleukin 10, and transforming growth factor-beta1 in multiply injured blunt trauma victims.. Crit Care Med.

[27] Conti B, Tabarean I, Andrei C, Bartfai T (2004). Cytokines and fever.. Front Biosci.

[28] Warren MA, Shoemaker SF, Shealy DJ, Bshar W, Ip MM (2009). Tumor necrosis factor deficiency inhibits mammary tumorigenesis and a tumor necrosis factor neutralizing antibody decreases mammary tumor growth in neu/erbB2 transgenic mice.. Mol Cancer Ther.

[29] Reed JR, Leon RP, Hall MK, Schwertfeger KL (2009). Interleukin-1beta and fibroblast growth factor receptor 1 cooperate to induce cyclooxygenase-2 during early mammary tumourigenesis.. Breast Cancer Res.

[30] Jiang S, Zhang HW, Lu MH, He XH, Li Y (2010). MicroRNA-155 functions as an OncomiR in breast cancer by targeting the suppressor of cytokine signaling 1 gene.. Cancer Res.

[31] Nowacki MP, Szymendera JJ (1983). The strongest prognostic factors in colorectal carcinoma. Surgicopathologic stage of disease and postoperative fever.. Dis Colon Rectum.

[32] Conze D, Weiss L, Regen PS, Bhushan A, Weaver D (2001). Autocrine production of interleukin 6 causes multidrug resistance in breast cancer cells.. Cancer Res.

[33] Zhang GJ, Adachi I (1999). Serum interleukin-6 levels correlate to tumor progression and prognosis in metastatic breast carcinoma.. Anticancer Res.

